# Vocabulary Matters: An Annotation Pipeline and Four Deep Learning Algorithms for Enzyme Named Entity Recognition

**DOI:** 10.1021/acs.jproteome.3c00367

**Published:** 2024-05-11

**Authors:** Meiqi Wang, Avish Vijayaraghavan, Tim Beck, Joram M. Posma

**Affiliations:** † Section of Bioinformatics, Division of Systems Medicine, Department of Metabolism, Digestion and Reproduction, Imperial College London, London W12 0NN, U.K.; ‡ UKRI Centre for Doctoral Training in AI for Healthcare, Department of Computing, Imperial College London, London SW7 2AZ, U.K.; § School of Medicine, University of Nottingham, Biodiscovery Institute, Nottingham NG7 2RD, U.K.; ∥ Health Data Research (HDR) U.K., London NW1 2BE, U.K.

**Keywords:** biomedical natural language processing, deep learning, named entity recognition

## Abstract

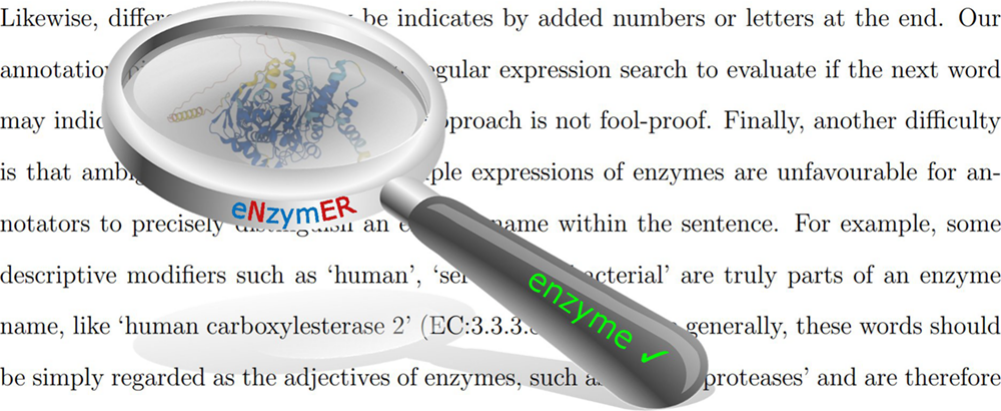

Enzymes are indispensable in many biological processes, and with biomedical literature growing exponentially, effective literature review becomes increasingly challenging. Natural language processing methods offer solutions to streamline this process. This study aims to develop an annotated enzyme corpus for training and evaluating enzyme named entity recognition (NER) models. A novel pipeline, combining dictionary matching and rule-based keyword searching, automatically annotated enzyme entities in >4800 full-text publications. Four deep learning NER models were created with different vocabularies (BioBERT/SciBERT) and architectures (BiLSTM/transformer) and evaluated on 526 manually annotated full-text publications. The annotation pipeline achieved an *F*1-score of 0.86 (precision = 1.00, recall = 0.76), surpassed by fine-tuned transformers for *F*1-score (BioBERT: 0.89, SciBERT: 0.88) and recall (0.86) with BiLSTM models having higher precision (0.94) than transformers (0.92). The annotation pipeline runs in seconds on standard laptops with almost perfect precision, but was outperformed by fine-tuned transformers in terms of *F*1-score and recall, demonstrating generalizability beyond the training data. In comparison, SciBERT-based models exhibited higher precision, and BioBERT-based models exhibited higher recall, highlighting the importance of vocabulary and architecture. These models, representing the first enzyme NER algorithms, enable more effective enzyme text mining and information extraction. Codes for automated annotation and model generation are available from https://github.com/omicsNLP/enzymeNER and https://zenodo.org/doi/10.5281/zenodo.10581586.

## Introduction

Enzymes are proteins that regulate biochemical reactions by decreasing the activation energy required for the chemical conversion of a substrate to a product. The largest online enzyme database, BRENDA,^1^ contains over 100 K different enzyme names that are obtained by manual extraction from literature, semiautomatic text mining, or by integration from other sources and is growing exponentially.^2^ Manual extraction of information from large numbers of publications is time-consuming and labor-intensive and may lead to human errors. With the biomedical literature expanding at an increased rate,^3^ there is a clear need to develop new methods to automatically identify and extract enzyme names from the biomedical scientific literature.

Natural language processing (NLP) is a subfield of artificial intelligence that uses statistics-based techniques to understand human language and has multiple different applications such as text prediction, classification, summarization and translation, and speech recognition.^4^ Recent advances in the field have resulted in the high performance of various NLP tasks with deep learning (DL) models and domain-specific word embeddings. Named entity recognition (NER) is an NLP task that locates and identifies specific entities in texts and classifies them into predefined categories. In recent years, NER models using DL models have flourished given their high performance on many tasks,^5^ using DL architectures such as bidirectional long–short-term memory (BiLSTM)-based recurrent neural networks^6,7^ and transformers.^8−11^ There has been much recent success of biomedical natural language processing (bioNLP) such as for gene/protein, disease, and chemical entity recognition with fine-tuned pretrained language models,^12−15^ and for reading clinical notes in electronic health records.^16^

The concept of pretraining refers to initializing network weights based on a large-scale corpus (a collection of texts/sentences). This is done to generate distributed representations of words or N-dimensional vectors as inputs for subsequent NER models. In NLP, this processing is known as word embedding which is a type of pretrained language model. The word embedding process can not only yield machine-understandable expressions of real words but also capture semantic and syntactic properties of a token which might not be explicitly present without word embedding,^5^ and this is context- and domain-dependent. Until a few years ago, static word embedding such as Word2Vec^17^ and GloVe^18^ was predominantly used. These have now been replaced by contextualized word embeddings that enhance the DL model's performance^19^ by focusing on neighboring words. These pretrained models can then be fine-tuned (changing network weights) for different downstream NLP tasks using transfer learning.^5^ The choice of pretrained model has a big influence on the final performance because general domain data sets, such as those to train Bidirectional Encoder Representations from Transformers (BERT)^9^ (trained on Wikipedia and Google Books corpus), may not contain the vocabulary and word embeddings that are necessary to process text from the biomedical literature.^19^ BioBERT is a biomedical language representation model that was pretrained on biomedical domain corpora (PubMed abstracts with 4.5B words and PubMed Central (PMC) full-text publications with 13.5B words) after initialization with BERT-weights and can be fine-tuned for NER, relation extraction, and question answering text-mining problems and has shown state-of-the-art performance for several NER tasks.^12^ SciBERT^11^ is a related model which was initialized from scratch and trained on full-text publications from the Semantic Scholar platform^20^ and thus does not inherit any vocabulary from BERT, making it potentially more useful for bioNLP tasks, as it was trained on scientific publications from the biomedical domain (82%) and computer science (18%).

Training of models for biomedical NER relies on the availability of biomedical text corpora in machine-readable format.^21^ Most corpora used for training (and evaluating) bioNLP algorithms are those either curated by the BioCreative committee or NCBI team and contain annotations for different entities, including for diseases,^22,23^ genes/proteins,^24−26^ mutations,^27^ and chemical compounds.^23,28,29^ The availability of corpora dictates the developments made in the field of bioNLP with mostly incremental gains over prior methods. No corpus exists that is specific for our task of enzyme NER; thus, only corpora with annotated protein entities can be used. However, the specificity is a problem since not all proteins are enzymes; thereby, training on protein corpora for enzyme recognition will inflate false positives. Moreover, the existing corpora with protein (and other) annotations come mostly from abstracts, as can be seen from the data used by CollaboNet.^30^

In this article, we aim to fill this gap by (1) describing a generalizable, dictionary-based annotation workflow to create an annotated corpus curated from full-text publications in a machine-readable format for enzyme NER, (2) contributing four DL-based models trained using two different architectures (BiLSTM and BERT) and two different word embeddings (BioBERT and SciBERT) to evaluate how vocabulary matters for NER, and (3) evaluating the performance on a manually annotated test set to compare the annotation pipeline and four DL models.

## Materials and Methods

### Data—Publications

The corpus was created from PMC Open Access publications from four different omics domains: genome-wide association studies (GWAS), proteomics, metabolomics, and microbiome. The GWAS and metabolomics publications that were used we described previously,^31,32^ the proteomics and microbiome publications were acquired in a similar manner as the metabolomics publication.^32^ For proteomics, the field keyword search terms were “proteomics”, “proteome”, “proteomic”, and “proteome-wide”, and the sample keyword search terms were “urine”, “urinary”, “blood”, “serum”, “plasma”, “faecal”, “faeces”, “fecal”, “feces”, “stool”, “cerebrospinal fluid”, “CSF”, and “biofluid”, with similar disease search terms. For the microbiome corpus, OA publications relating to airway, fecal, skin, urinary, and vaginal microbiomes were searched for with additional keywords such as ″16S”, “rRNA”, “sequencing”, “shotgun”, “Illumina”, “MiSeq”, and “PCR”.

The publications were converted from HTML, standardized, and converted to the machine-readable BioC-JSON format using Auto-CORPus.^31^ First, Auto-CORPus converts the main text of each publication from HTML to the BioC JSON format. In this file, the pipeline splits the publications into different sections using the Information Artifact Ontology (IAO),^33^ and in each section, each paragraph forms a single “text” feature. Second, Auto-CORPus transforms tables inside publications to a table-JSON format, and it extracts abbreviations from both the main text and separate abbreviations sections (if available) within the main text and generates another JSON file for abbreviations with linked full definitions. Here, we use the main text (BioC-JSON) and abbreviation JSON files for preprocessing and to build the data sets for DL models.

### Data—Enzyme Nomenclature

A list of enzyme codes with names and synonyms was downloaded on 28/10/2021 using the KEGG REST API (https://www.kegg.jp/kegg/rest/keggapi.html). This list contains over 28,000 enzyme names/synonyms that were used for dictionary matching. Several changes were made to this dictionary to expand it to improve its ability to identify matching entities. Missing entities were added that have the same meaning but different spelling styles compared to other items within the list. For example, some enzymes connect different words using hyphens, while others use spaces instead, and others use Greek letters instead of spelling these out using the Latin alphabet. Therefore, additional entries were created in the dictionary to account for these differences and capture more entities when matching the texts.

### Annotation Pipeline

The dictionary was further adapted to accelerate the searching process. We reconstructed the dictionary list in a novel hierarchical format by clustering entities with the same features into smaller streams based on enzyme classes. We performed this based on an analysis of the enzyme nomenclature, where most (but not all) enzyme names end in “ase”. We use the seven categories of enzymes based on the different chemical reactions in which they are involved in. For example, enzymes involved in oxidation reactions are likely ending in “oxidase”, whereas “reductase” enzymes perform reduction reactions. Using this structure, we classify the items within the list in a tree-structured (hierarchical) dictionary. In that way, when searching through the text, the pipeline can use regular expression (RegEx) rules to search the node-words from the root of the word to determine which branch-list to use to speed up the searching and autoannotation process.

Supervised NER algorithms rely on annotated corpora; however, none exist for enzymes. We created an enzyme-specific corpus using a fast automated pipeline for annotation using a hierarchical dictionary of enzyme terms. The pipeline uses rule-based RegEx to assist with the search process of potential entities in full-text articles. The BioC JSON files from Auto-CORPus were used as input to the annotation pipeline, with the enzyme entities in each paragraph added to the “annotations” section of the JSON document, which includes a unique id, an external identifier (EC number) if a direct match is found (if no direct match is found, this is replaced by the root word), and the location in the text (based on the offset of the paragraph in the entire article).

The pipeline consists of two search steps to find the entities. First, there are direct matches of dictionary terms in the text (an exact match to a known entity). Second, keyword searching using root enzyme terms (e.g., transferase) to perform rule-based fuzzy matching to identify entities that do not directly match to elements from the dictionary. Specifically, the pipeline utilizes the spaCy package (v3.2) to split each paragraph into individual sentences, in which each word is contrasted with patterns created by the searching rules based on the structure of the enzyme dictionary.

If a root word is matched, then the pipeline will first search throughout the given node in the list for a content-matched item (the first step). In this step, the greedy algorithm was used to ensure that the matched entity is the one with the maximum length in this sentence. For example, the term ‘aliphatic alcohol dehydrogenase’ also contains other matches to terms “alcohol dehydrogenase” and “dehydrogenase”, and this approach ensures any terms contained within another are not selected.

If no direct match is found, then the pipeline continues with step 2. The principle of partial searching is that if a word is regarded as a part of an enzyme name, such as a word that is matched with a node-word in the dictionary, and words ending with “ase” are considered as a part of a potential enzyme. The next steps consider the words preceding and following the identified entity and evaluate if these are part of the enzyme entity. In this step, common words ending in “-ase”, e.g., “phase” and “database”, are ignored. To establish the number of words before and after an entity to consider, we analyzed the known enzyme names in the dictionary. Over 92% of enzyme entities in the list end in “-ase”, and from these, over 99% consist of five words or less, indicating that we can restrict our search to four words before the identified “ase”-word. For words after the entity, we use additional RegEx rules as most of the contents after the “ase”-word are enclosed in brackets, but cases exist where there is a single number or Greek letter. Therefore, we created rules based on these features to support backward searching and to add these to the identified entity.

To increase the number of training cases, we also include sentences in which abbreviations of enzymes appear. We applied the pipeline to the list of definitions (abbreviations output JSON file from Auto-CORPus) in the article, and the abbreviations of any enzymes identified are then searched in the main text. These abbreviations are annotated in the same way as full terms; however, for training, these are replaced by their full definitions. Abbreviations are not used by themselves, as this can increase the number of false positives, as some abbreviations can have different definitions in different articles.

### Training, Validation, and Test Splits

The corpus of 3,525 full-text publications that contained any enzyme mention was split 75:10:15 into training:validation:test sets with each portion containing an equal proportion of GWAS, proteomics, metabolomics, and microbiome articles (Table 1). The training and validation sets were used to construct the DL models, with the test set separately undergoing manual annotation and used to evaluate the performance of the DL algorithms. The training and validation sets were annotated using the annotation pipeline.

**Table 1:** Summary of the Enzyme Corpus

domain	publications (*n*)	training set (*n*)	validation set (*n*)	test set (*n*)
GWAS	884	664	88	132
proteomics	446	336	44	66
metabolomics	1006	756	100	150
microbiome	1189	893	118	178

Only the sentences containing enzyme entities constitute the data set for training models. Therefore, we extracted those annotated sentences to build up the final corpus for DL models. Each sentence gets a unique ID which consists of the PMCID (i.e., paper ID with PMC identifier), a sentenceID that determines which section (e.g., Results) and paragraph the sentence belongs to. Unlike our metabolite NER model^32^ where abbreviations of metabolites were kept in, here we replaced all abbreviations in the full text with the definitions from the abbreviation output files. To increase the number of annotations for training, we consider not only the textual abstract, methods, results, and discussion sections (as done for metabolite NER), but we also include the introduction and references sections as a supplement.

**Figure 1:**
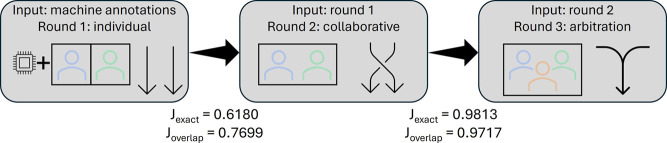
Graphical description of the test set annotation process.

The test set consists of 526 full-text publications. These were annotated in a three-stage process (Figure 1). First, the articles were annotated using the automatic annotation pipeline (see above) and uploaded to a local installation of TeamTat,^34^ followed by two annotators independently annotating the articles and verifying the machine annotations. The annotators were given examples of how enzymes are described in the KEGG database. Second, after finishing all articles, the two annotators worked in collaborative mode in TeamTat to resolve disagreements, add missing concept identifiers (EC numbers) where possible, and annotate missing entities. The final step was also done in collaborative mode, where conflicts between annotators indicated by TeamTat were resolved with a third annotator (arbiter). Conflicts included cases where only one annotator has annotated an entity, where annotated entities are overlapping, and where the concept ID is different between annotators.

### DL Models

In this study, we use two network structures to compare their performance, for which the parameters are described in the sections below. First, we use a BiLSTM network due to its prior demonstrated state-of-the-art performance for chemical NER tasks in the CEMP BioCreative V.5 challenge^35^ with ChemListem,^36^ a type of BiLSTM model, as well as for metabolite NER with MetaboListem and TABoLiSTM^32^ that are based on ChemListem. The latter (TABoLiSTM) replaced the GloVe embedding method with BERT^9^ and BioBERT.^12^ Here, we use the same BiLSTM model but with BioBERT^12^ (as previous) and SciBERT^11^ for the word embedding layer. Second, we fine-tune the BioBERT and SciBERT models as transformer models (without BiLSTM) for the NER task. All models were trained (for 10 epochs) on the data set we created with the annotation pipeline, with each epoch trained in mini batches on a Linux workstation with an Intel Core i7 CPU (10700K, 3.8 GHz, 8 Core) with 32GB RAM and a 10GB NVIDIA GeForce RTX 3080 GPU with 8,704 CUDA parallel-processing cores. The epoch with the highest *F*1 score for the validation data set was stored and used to evaluate the gold-standard test set (i.e., manually annotated data set). Each of the 4 models was trained with 10 different random states to compare score distributions as recommended by Reimers and Gurevych.^37^

#### Transformer as Tokenizer of BiLSTM Network

We used biobert-base-cased-v1.2 and scibert_scivocab_cased from Huggingface^38^ as transformer-based word tokenizers to generate the vocabulary and corresponding word embedding architectures used in the BiLSTM NER models. Here, the SOBIE system was applied to mark each token as a specific tag; in which ‘O’ (for outside) labels a token that is not part of an entity, ‘S’ refers to singleton that marks a one-word token (i.e., the whole of an entity), and ‘B’ (beginning), “I” (inside), and ‘E’ (end) mark a multiword token.

The BiLSTM model contains two inputs that are concatenated; the first input layer is a Conv1D with a dropout of 0.5 to process the name-internal features from a preclassifier; this is a convolutional layer with 256 neural dimensions. The preclassifier subsystem was implemented as in prior work^32,36^ using a random forest. The preclassifier works by generating name-internal binary features that are either “O’ or ‘SBIE” labels and assigns a probability to each token for it to be part of an enzyme name (S, B, I, or E). These tokens are then segmented by BioBERT or SciBERT, respectively, to match the corresponding models. Afterward, these pretrained features are passed to the BiLSTM network. The second input to the BiLSTM network is the features from the word embedding layer (here BioBERT and SciBERT are separately applied to this layer), where text tokens are converted to a sequence of integers. These contextual embeddings were initialized by pretrained weights of the transformer models with a 768 hidden dimensional size, feeding the output from the embedding model through a SpatialDropout1D layer with a dropout rate of 0.1 to prevent overfitting.

Both input layers are concatenated to result in a 1024-dimensional vector (256 + 768) as the final input into the BiLSTM network that contains a 64-dimensional output space. The activation function selection is with a tanh (hyperbolic tangent) with a sigmoid activation function applied to the recurrent step of the network. The regularization penalty used for the kernel weight matrix was l1_l2 in which both the l1 and l2 losses are limited up to 1 × 10^–6^. The output layer is a time-distributed dense layer with a softmax activation function to ensure the sum of the 5 output probabilities (‘O’, ‘S’, ‘B’, ‘I’, and ‘E’) for each token totals to 1.

#### Fine-Tuned Transformers

We used two transformer models (using the respective tokenizer) from Huggingface (dmis-lab/biobert-v1.1 and allenai/scibert_scivocab_uncased) to fine-tune models on the same training corpus. These models were trained with the BIO system (Beginning, Inside, Outside) using cross-entropy loss for training and validation, with Adam as optimizer, a learning rate of 1 × 10^–4^, warmup steps of 0.1, dropout of 0.3, and with gradient accumulation every 4 steps.

To the best of our knowledge, our work is the first time that algorithms are developed for an annotation pipeline and NER models specifically for enzymes (opposed to all proteins). We compare our enzyme NER results with the BERN2 multitask model,^15^ which is based on BioBERT, for the gene/protein entity tag, as no enzyme-specific NER model currently exists. We therefore evaluated the performance of the BERN2 model on our manually annotated enzyme corpus and compared it with our enzyme-specific models. The BERN2 model can be accessed remotely through a web API (http://bern2.korea.ac.kr/), but this is limited to 300 requests per 100 s per user. For a quicker turnaround time on our large corpus, we followed the authors’ guidelines in the BERN2 repository (https://github.com/dmis-lab/BERN2) to install and run a local version of the model on a Linux workstation. Some slight modifications were made to get the model running properly, which we describe in the Supporting Information. Once the BERN2 model is running on a local port within its conda environment, we make requests to it using the URL associated with the port. Running our corpus through the model returns a set of found entities per paper (and optionally per paragraph), which fall into one of nine biomedical classes: gene/protein, disease, drug/chemical, species, mutation, cell line, cell type, DNA, and RNA. We filter entities for the gene/protein class to enable protein NER comparison to our model.

#### Applicability of Our Model to Improve Metabolite NER

The metabolite NER^32^ and enzyme NER tasks were carried out independently and hence used different training, validation, and test sets. To compare the output of both algorithms, we evaluated the overlap of the two test sets, yielding a total of 18 metabolomics publications that have not been used as part of the training or validation stages in either algorithm. We extracted all sentences with manually annotated metabolites and/or enzymes and compared the outputs of both NER algorithms to evaluate whether our model can help to improve the accuracy of the metabolite NER output.

### Evaluation Metrics

To evaluate the interannotator agreement (IAA), we calculated the Jaccard index using the number of agreed upon entities between annotators (“AB”) in the numerator and the total number of annotations (including those only made by one annotator, “A’ and ‘B”) in the denominator (eq 1). Cohen’s kappa was not used as this includes true negative annotations, of which there are many in NER tasks. We evaluated the IAA in two ways: at the entity and character levels. The entity level is an exact measure, where both annotations must match exactly. The character level is an overlap measure, where partial overlap (see below) is considered.
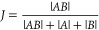
1

To evaluate the performance of the annotation pipeline and DL models, we calculated 3 properties of a confusion matrix: true positive (TP) entities, false positives (FPs), and false negatives (FNs). FPs are entities predicted to be an enzyme by a model that does not match an entity in the test set. FNs are entities that were not annotated by the model but are contained in the test set annotations. Using these measures, we calculate the precision (probability of correctly predicted positive values in the total predicted positive outcomes, eq 2), recall (also known as sensitivity, probability of correctly predicted positives relative to all true positive cases, eq 3), and *F*1-score (weighted average of precision and recall, eq 4). Here, we first converted the SOBIE and BIO tags to “entity” (SBIE; BI) and “nonentity” (O). Correctly identified ‘O’ tags, the majority of tokens were not included in the calculation of the precision, recall, and *F*1-scores. These are only based on the tokens predicted as entities by the algorithms or tokens that were annotated as entities in the gold-standard test set. Since not all proteins are enzymes, and hence there can be many false positives, we evaluate BERN2 using the recall only (we do present the precision and *F*1-score for completeness).
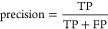
2
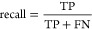
3
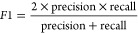
4

Partial positive (PP) matches, i.e., where the only root term is correctly identified or where the indication of an isoform is missing but the remainder of an entity is correctly annotated, were separately tracked, and the final count of PPs was split equally between TP and FN.

## Results and Discussion

### Corpus Creation and Hierarchical Dictionary

**Figure 2:**
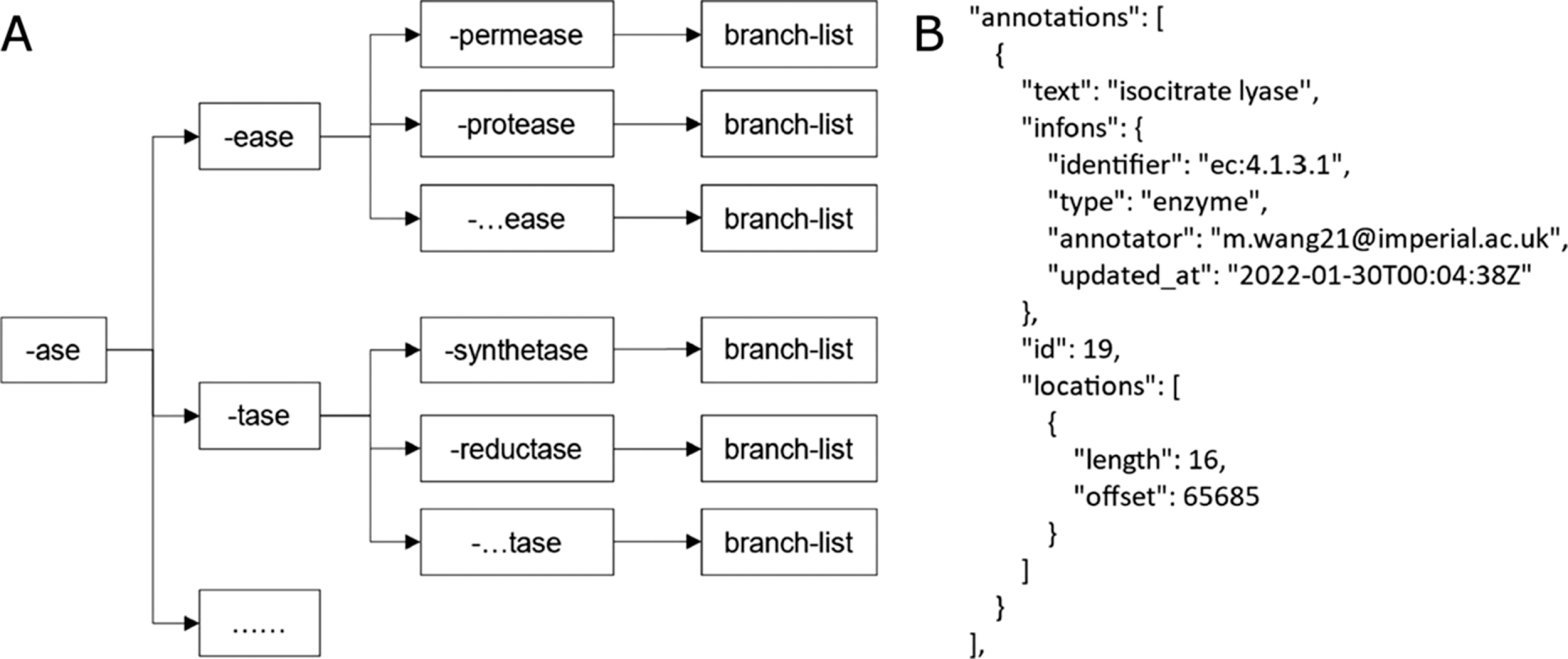
(A) Example of the hierarchical structure of the dictionary showing a part of the “-ase” branch. Enzymes not ending in “-ase” are contained in the dictionary in other branches. (B) Example of annotation format as an individual dictionary displaying the detail of an entity with four elements (i.e., “text”, “infons”, “id”, “locations”) to represent a unique entity in the text. Where offset indicates the starting character of the annotation in the full text.

The reconstructed dictionary list, using the hierarchical format (Figure 2A), speeds up the searching process compared with a standard dictionary search. An entire full-text article can be annotated in 0.5s using this approach, compared to 1s per file using the standard approach, due to the reduction in size of the branch list resulting in less evaluations.

Our annotation pipeline was used to create an annotated corpus for supervised learning. The enzyme entities in the texts are extracted and coded into the “annotations” section of the BioC JSON file (output of Auto-CORPus) that follows the text content of each paragraph; see example in Figure 2B. Each annotation has a number of key-value pairs: “text” is the name of the identified entity, “infons” contains the information on the identifier (E.C. number or keyword), type (enzyme), annotator, annotation time, “id” is a unique ID for each annotation in the text and indicates the order in which the entity is annotated, and “locations” indicates the position of the annotation within the full text.

The annotated corpus is easy to use and can be reused in the future to convert the text and annotations to different formats to be used for training different algorithms. The JSON structure also lends itself to contain multiple entity types, which means multitask learning corpora can be prepared for biomedical NER tasks. For domains without annotated corpora, but where there exists some level of hierarchy and/or “root” words common among entities, our automated annotation approach may provide a useful first step to create such data for training. For example, the hierarchical annotation approach may also be applicable for speeding up the process of annotating other biomedical entities such as pathways, gene names, or metabolites. For example, pathways also have a similar nomenclature as enzymes in which entities end in similar ways such as the root words “synthesis”, “biosynthesis”, “degradation”, and “metabolism”, and at present, no such corpus exists and can be created in a similar manner.

However, our pipeline includes some modifications to improve the accuracy and effectiveness of the annotation. The weakness of the annotation pipeline mainly lies in the second searching step (i.e., keyword searching). Once a root word has been identified, the preceding and following words are evaluated to determine whether these are part of the enzyme. The dictionary contains several spellings of an enzyme; however, different forms may still exist that may include (or exclude) the use of hyphens, which will prevent terms from being matched directly. Likewise, different isoforms may be indicated by added numbers or letters at the end. Our annotation pipeline uses a rule-based regular expression search to evaluate if the next word may indicate an isoform; however, this approach is not fool-proof. Finally, another difficulty is that ambiguous semantics and multiple expressions of enzymes are unfavorable for annotators to precisely distinguish an enzyme name within the sentence. For example, some descriptive modifiers such as “human”, “serum”, or “bacterial” are truly parts of an enzyme name, like “human carboxylesterase 2” (EC:3.3.3.84), but more generally, these words should be simply regarded as the adjectives of enzymes, such as “human proteases”, and are therefore not included in our annotations unless for some cases where there are exact matches. The difficulty of manual annotation highlights how this process may be replaced with an algorithm before being subjected to human verification.

Our training data set created from the annotated JSON files additionally replaces all abbreviations of enzymes by their definitions; hence, the evaluation of abbreviated entities relies on correctly identifying abbreviation-definition pairs. Our annotation pipeline annotated over 30,000 entities in over 3,000 articles (Table 2). As expected, the proteomics articles have the largest average number of entities (23), followed by metabolomics (9) and GWAS (8). Across all annotated entities, the most common single word annotation is polymerase, whereas when including multiword entities, the most common root terms are kinase and protease (Table 3). No enzyme-specific corpus exists, and our corpus is also larger than most bioNLP corpora.^39^

**Table 2:** Summary of the Annotated Enzyme Corpus per Biomedical Domain, the Total Number of Enzymes Annotated, and the Mean and Standard Deviation across Articles

domain	annotated enzymes	mean ± st. dev.
GWAS	6736	8 ± 17.77
proteomics	10,037	23 ± 29.30
metabolomics	9447	9 ± 13.76
microbiome	5502	5 ± 7.88
total	31,722	9 ± 17.11

**Table 3:** Number of Components of Entities Identified by Keyword Searching for the 10 Most Found Root Terms

root term	single word	2 words	3 words	3+ words	total
kinase	631	446	201	74	1352
protease	661	452	108	27	1248
polymerase	1017	140	27	13	1197
synthase	53	107	257	105	522
dehydrogenase	137	232	36	17	422
metalloproteinase	117	178	24	2	321
phosphatase	68	65	83	25	241
reductase	98	97	37	8	240
transferase	71	83	57	24	235
hydrolase	64	134	25	9	232

### Model Evaluation

The test set was first machine annotated and subsequently verified by two human annotators over multiple stages (round one individual/independent and round two in collaborative mode). After the first round (annotators can only see the machine annotations), there was an IAA of 0.62 at the entity level (exact) and 0.77 at the character level (overlap); see Figure 1. The second collaborative round (where annotators can see each other's annotations) showed many of the disagreements were due to one annotator systematically including suffixed numbers (isoforms) and another annotator only including these if these were present in the ontology. After the second round, the IAA increased to 0.98 for the exact and 0.97 for the overlap measures. The final step used an arbiter to settle on remaining disagreements; however, in practice, the disagreements in the final step only included cases where an entity (already annotated elsewhere in the article) was missed by one annotator but not by the other and therefore easy to resolve.

As described above, we tracked all partial matches and split these between TPs and FNs for the different algorithmic models. We also analyzed the list of PP items. Some of these PPs are similar (but not identical) between the manually annotated test set and the annotation (or DL) pipeline. The main differences related to a capital letter or a number not captured by the annotation pipeline (i.e., isoforms). Most other differences relate to the method only identifying the root term (“kinase” instead of “Ser/Thr kinase”). The former is a minor mistake (could be considered a TP), whereas the latter clearly misses an important part of the enzyme (and perhaps should be a FN). Resolving PPs into TPs/FNs would involve a fourth independent person (from the annotators and arbiter) to go over each of these entries, which was not feasible given the time/effort required. Therefore here, we regarded half of the PPs as TP and the other as FN.

The precision of the pipeline is effectively 1.00 (see Table 4), and there is only one false positive (“protein A”). However, the lower recall of 0.76 demonstrates that there are certain enzyme entities that were not found by the annotation pipeline (a higher degree of FNs).

**Table 4:** Performance Metrics (Expressed as Percentage) for Different Models Evaluated on the Manually Annotated Test Set (*n* = 526 Full Text Articles)^a^

model	embedding	precision	recall	*F*1-score
annotation pipeline		**0.996**	0.762	0.863
BioBERT-BiLSTM	BioBERT^12^	0.937 ± 0.015	0.759 ± 0.034	0.838 ± 0.016
SciBERT-BiLSTM	SciBERT^11^	*0.943 ± 0.006*	0.748 ± 0.018	0.834 ± 0.009
BioBERT	BioBERT^12^	0.916 ± 0.003	**0.861** ± **0.005**	**0.888** ± **0.002**
SciBERT	SciBERT^11^	0.921 ± 0.005	*0.858 ± 0.010*	*0.881 ± 0.011*
BERN2^15^	BioBERT^12^	0.148	0.824	0.250

a The average and standard deviation for 10 random seeds are given for the BiLSTM and fine-tuned models. The highest and second highest evaluation metrics are given in **bold** and *italic*, respectively.

As an alternative to dictionary searching combined with rule-based annotation, we previously created two DL models based on the BiLSTM architecture that achieved state-of-the-art results in metabolite NER.^32^ Here, we extended that work in the context of enzyme NER by comparing the best embedding found previously (BioBERT^12^) with the newer SciBERT^11^ model which includes its own vocabulary. In addition, here, we include the fine-tuned versions of BioBERT and SciBERT as well to compare with the BiLSTM-based models.

The highest precision is achieved by the annotation pipeline (Table 4), followed by the SciBERT-BiLSTM model. Both BiLSTM-based models outperform the fine-tuned transformer models in terms of precision (0.94 vs 0.92). However, the highest recall is achieved by the fine-tuned transformer models with 0.86 for both BioBERT and SciBERT, considerably higher than the annotation pipeline and BiLSTM-based models (0.75–0.76). Overall, both fine-tuned transformer models outperform the BiLSTM models as well as the annotation pipeline with an *F*1 score of 0.89 for BioBERT and 0.88 for SciBERT, with BioBERT models showing less variability than SciBERT models across the 10 random seeds. This highlights the benefit of using DL algorithms for NER over (traditional) dictionary-based approaches, where a lower precision for DL methods is accompanied by a substantially higher recall.

Intuitively, we would expect that the BioBERT-based models would yield better performance than SciBERT ones, since they were specifically pretrained on a biomedical corpus, with SciBERT trained on a corpus that contains over 80% of papers from the broad biomedical domain supplemented with 20% computer science papers. SciBERT constructed a new vocabulary, SCIVOCAB, for tokenization and word embedding, which is substantially different compared to the WordPiece vocabulary used by BERT and inherited by BioBERT (which was built on top of BERT). While Beltagy et al. indicate that the overlap between the two vocabularies of tokens is a mere 42%, and SCIVOCAB providing more detailed tokens with biomedical features,^11^ our results do not show significant differences between SciBERT and BioBERT for the enzyme NER task.

With the development of biomedical NLP, more and more DL architectures are built or pretrained as transformer models, as we have done here. BioBERT has been reused by the same authors to create a model (BERN2^15^) for joint NER and named entity normalization for 9 different entities including proteins (achieving an *F*1-score of 0.84 on the BC2GM corpus^24^). BERN2 achieves a recall of 0.82, clearly outperforming our dictionary-based annotation pipeline and the BiLSTM models but not reaching the same level as our dedicated enzyme fine-tuned transformer NER models (0.86).

Others have used large-language models (LLMs), such as GPT,^10^ for biomedical NLP tasks with zero- and few-shot learning with varying levels of success, demonstrating that LLM outperforms BERT-based methods on question-answering tasks, but BERT-based models considerably outperforming different LLMs for classification, relation extraction, natural language inference, and NER.^40^ Improving LLMs for NER tasks requires at least further fine-tuning but more likely supplying these with domain-specific training data for domains that they are not trained on. In our case, not all biomedical literature is freely shareable, and it is therefore not possible to send these data to external platforms to train such models, a problem that is potentially solvable by generating synthetic data for closed systems.^41^ Another issue is available compute to train such models that, even with open LLMs such as LLaMa,^42^ require much more resources to train than BERT-based models. We describe here an approach to generate training data for a new domain, addressing one of the limitations of BERT over LLMs in requiring task-specific training data. BERT-based models have another advantage over LLMs due to the bidirectional, opposed to autoregressive (generative), design allowing for more fine-grained contextual understanding required for biomedical NER tasks.

Our annotated data set contains over 30,000 entities (Table 2) compared with approximately 20,000 for the BC2GM corpus.^24^ While the protein NER corpora will include enzymes in the training data, our training set is much larger, which means that the models can be trained with more data relevant to the task; the same goes for the number of enzymes in the test sets when comparing our corpus to the protein-specific corpora. Moreover, the methods used here for enzymes are amenable to any type of protein to facilitate the creation of a multitask protein NER model and other biomedical text-mining tools.

We used two labeling schemes for the data, SOBIE for the BiLSTM models (to allow us to use the preclassifier as previously defined) and BIO for the fine-tuned transformers. In order to compare the performance of each model, we converted the output (SOBIE/BIO) to either belonging to an entity or no entity, with the evaluation of model performance only considering the tokens that are entities either in the manually annotated test set or entities annotated by any of the 5 models. Most NER tasks make use of the BIO format (including BioBERT,^12^ SciBERT,^11^ and BERN2^15^ as used here); however, the SOBIE format has a benefit for tasks for which there exist multiword discontinuous entities. However, the level of discontinuity was almost nonexistent in our corpus; therefore, we did not explore this further for the task of enzyme NER as any gains were considered to be marginal, but this may be more relevant for other domains such as in electronic health records.^43^ We assigned partial matches equally between TP and FP/FN (as appropriate), this is following the recommendations from the Message Understanding Conference (MUC).^44^ The MUC introduced several other measures, such as mismatches between identifiers (with entities being correct), that are more relevant for named entity normalization tasks. While these metrics give a better representation of the errors and performance, they are not commonly used in the evaluation of modern NER models and, as such, would not make it possible for us to compare our results with models such as BERN2.

### Model Application to Improve Metabolite NER

Our prior work^32^ showed that some entities recognized as metabolites are actually part of enzyme names and hence ought to be considered as false positives when evaluating these in the context of metabolite NER. Our work here on enzyme NER can be used to address this limitation by filtering out any metabolite entities that overlaps with an enzyme entity.

We show in Table 5 several examples of applying both TABoLiSTM and SciBERT-BiLSTM (the DL model with the highest precision) to the same sentence. We have repurposed the TABoLiSTM metabolomics corpus (over 1,000 publications in that corpus contain at least 1 enzyme, see Table 1) in this work; therefore, in order to evaluate how DL models can improve TABoLiSTM, we only give examples from articles that were part of either test set. Table 5A contains examples where we identified no benefit of using enzyme NER models to improve the output of TABoLiSTM as no entities overlap, hence TABoLiSTM correctly did not identify “tyrosine” as metabolite, as it was part of an enzyme name (it was not trained with this purpose). In Table 5B, there are several examples where TABoLiSTM reports a metabolite that is actually part of an enzyme (enzyme NER model output) such as identifying “ceramide” twice in a sentence where only one of these is a metabolite and the other is part of “ceramide synthases”, or identifying “deoxycytidine” as metabolite when our model identifies it as “deoxycytidine kinase”. Moreover, TABoLiSTM^32^ may also have an application to our enzyme NER work here, as it can be used to identify whether there is a metabolite name directly in front of a root term (in cases where only a root term is identified, which is what our annotation pipeline is highly effective in identifying). This demonstrates that these models can be used together to improve each other, while this can be done in the context of employing these algorithms in parallel, other work^15^ has shown that multitask learning will yield both better performance and small model sizes.

**Table 5:**
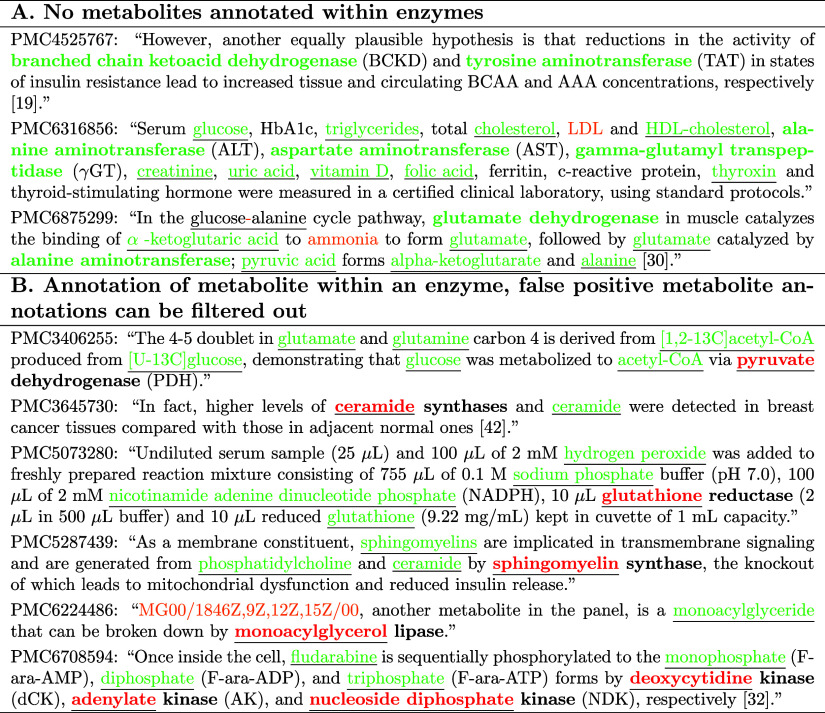
Example Output from Metabolite NER^32^ (Underlined) and Enzyme NER (in Bold)^a^

a Correct annotations are indicated in green, false positive metabolite annotations within enzymes in red, and false negative metabolite annotations in orange. For the purposes of diversity of metabolites and enzymes, we show one example per article to illustrate different cases. Additional examples are in the Supporting Information.

Likewise, there is potential to use multiple NER models as an ensemble, typically used for multitask learning (e.g., BERN2) but also single-task NER. For example, such an approach can make use of models with high precision (but lower recall) together with other models with high recall (but lower precision) as is the case here. This is beyond the scope of this work; however, a weighted voting scheme to aggregate results with a meta-classifier may result in higher precision and recall than any individual method can, as would a hierarchical scheme which first utilizes high recall methods and subsequently filter these with high precision methods.

Another potential avenue of future work involves expanding the training data by replacing abbreviations with their full definitions and thereby adding more training examples. This is possible with Auto-CORPus as it includes a separate list of identified abbreviations;^31^ however, this is still dependent on improving the accuracy of abbreviation detection algorithms. While many abbreviations are defined in a standard manner in which characters can be related to the definition that precedes or follows it at the first mention, there are also cases where there are nonexact mappings between abbreviations and definitions. For example, using a Greek letter to abbreviate the Latinized spelled out word (see gamma-glutamyl transpeptidase abbreviated as γGT in Table 5), added characters and/or discontinuity (F-ara-AMP) or using domain-specific knowledge to abbreviate entities (it is common in chemistry to abbreviate “hydroxy” to “OH”). Directly including abbreviations in training is not recommended unless these are unmistakable, since abbreviations can be domain-specific. A logical next step is to further improve abbreviation detection algorithms (currently using rule-based methods) by taking a DL approach to focus on relation extraction between abbreviations and definitions. Another aspect we identified is that punctuation and hyphenation also impact the performance of the annotation pipeline (e.g., “dipeptidyl peptidase-4” and “coenzyme A-linked enzyme” were not identified by the pipeline). Further improving the pipeline requires both programming new rule-based algorithms and a human-in-the-loop system to more effectively devise rules to improve the accuracy of the automatic annotation pipeline.

Finally, our training corpus contains a lot of positive examples. There are obvious advantages in including negative samples during training, as this may increase the generalizability and robustness of the models. However, this may also increase the noise during training and focus less on the verified patterns of entities in the data. This is relevant to enzyme NER since most enzymes end in “-ase”, only very few enzymes do not, and our hierarchical dictionary is able to successfully identify root terms (and distinguish these from other common -ase words such as database, case, phase, etc.).

## Conclusions

In conclusion, this work contributed a novel dictionary- and rule-based automatic pipeline for enzyme NER with almost perfect precision and which runs in a matter of seconds (on a standard laptop) on an entire full-text article because of the hierarchical dictionary. We used this pipeline to create a first-of-its-kind corpus for enzyme NER to facilitate text mining. As part of this corpus, we include a human-labeled test set. Our best performing model overall was the fine-tuned BioBERT model, and this DL model achieved an *F*1-score (0.89) that exceeded the silver-standard labeled training data (0.86) for enzyme NER, demonstrating a recall almost 0.1 higher than the annotation pipeline and BiLSTM models while sacrificing precision. The BiLSTM-based models did not surpass the training data in performance on any metric. Combining our proposed pipeline with DL models can facilitate more effective enzyme text-mining and information extraction research for literature review, and we demonstrated its use to improve other biomedical NER tasks.

The code for automatic annotation, model training and inference, as well as the data for training and testing are available via Github (https://github.com/omicsNLP/enzymeNER) as well as Zenodo (https://zenodo.org/doi/10.5281/zenodo.10581586).

## Supporting Information Available

Details of the installation of the BERN2 model; instructions for annotators; additional examples where no metabolites annotated within enzymes; and additional examples where metabolites annotated within enzymes (PDF)
