# Supplementary material for: Vocabulary Matters: An Annotation Pipeline and Four Deep Learning Algorithms for Enzyme Named Entity Recognition
Source: J Proteome Res. 2024 May 11;23(6):1915–25. doi: 10.1021/acs.jproteome.3c00367 (PMC11165580; doi:10.1021/acs.jproteome.3c00367)
Supplement: Supplementary file 1 — pr3c00367_si_001.pdf [file pr3c00367_si_001.pdf]

Supporting Information: Vocabulary Matters: An Annotation Pipeline and Four Deep Learning Algorithms for Enzyme Named Entity Recognition

Meiqi Wang,[1,5] Avish Vijayaraghavan,[1,2,5] Tim Beck,[*,3,4] and Joram M. Posma[*,1,4]

[1]*Section of Bioinformatics, Division of Systems Medicine, Department of Metabolism, Digestion and Reproduction, Imperial College London, W12 0NN, United Kingdom*

[2]*UKRI Centre for Doctoral Training in AI for Healthcare, Department of Computing, Imperial College London, SW7 2AZ, United Kingdom*

[3]*School of Medicine, University of Nottingham, Biodiscovery Institute, NG7 2RD, United Kingdom*

[4]*Health Data Research (HDR) UK, United Kingdom*

[5]*Contributed equally to this work*

*E-mail: tim.beck@nottingham.ac.uk; jmp111@ic.ac.uk

Supporting Information

- Supplementary Methods S1. Details of the installation of the BERN2 model.
- Supplementary Methods S2. Instructions for annotators.

- Supplementary Table S1. Additional examples where no metabolites are annotated within enzymes.
- Supplementary Table S2. Additional examples where metabolites are annotated within enzymes.

Supplementary Methods

S1. BERN2 installation

We describe setting up the BERN2 model on a Linux workstation with a GPU. In the "run_bern2.sh" script, we changed "python" to "python3". Every time it runs, BERN2 creates a "log/" directory to record run details which can help identify the location of a problem. From this, we found an issue with the installation of `GNormPlusJava` (the gene/protein normalisation tool) which required us to redownload an alternate version of the `CRF++` tool. BERN2 may try to access a port that is already being used - if the process using the port is not important, it can be killed using the following command `fuser -k [port number]/tcp`. If BERN2 is not running properly, it can be restarted cleanly by: deleting the "log/" folder, stopping BERN2 using the appropriate script, and deleting any CUDA artefacts.

S2. Guidelines for annotators

Both of our annotators were knowledgeable in terms of the enzyme nomenclature. They were trained to use the TeamTat software[S1] and viewed documents independently. Rather that start from scratch, they viewed documents annotated by our automated pipeline. This included identifiers for entities with exact matches to our dictionary entries (E.C. codes) or the root term identified. Both annotators were aware of the dictionary used, and of our changes made (removing prefixes such as 'human' and 'bacterial' when the ontology contained multiple synonyms). The ontology did not contain synonyms for all types of isoforms, and annotators were asked to include these in their annotations. Likewise, we initially asked

our annotators to add identifiers for the annotations they made, however this slowed down the task considerably, and it was decided to drop this aspect as this was not relevant for the NER task of this work. For this reason we did not use TeamTat's tools for reporting inter-annotator agreement as the metrics also require matching conceptID (i.e. identifiers) for a complete match.

After the first round of individual annotations (where they can only see the machine annotations), the annotators then worked in collaborative mode in which they could see the other person's annotations alongside their own. This process resolved the vast majority of conflicts and greatly improved the inter-annotator agreement. Any differences that remained were then identified and an arbiter set up to rule on these. However, in our case, after the second round the only differences were some missed annotations that were already annotated elsewhere in the document. We only worked with two annotators, but TeamTat is capable for multiple annotators to work simultaneously on the task.

Supplementary Tables

Table S1: Example output from metabolite NER[S2] (underlined) and enzyme NER (in bold). All sentences (in addition to the table in the main manuscript) for all 18 articles used for the evaluation (see Materials and Methods). Correct annotations are indicated in green, false positive metabolite annotations within enzymes in red, and false negative and other false positive metabolite annotations in orange. Note: formatting (e.g., superscript) is removed after processing with Auto-CORPus[S3] before NER. Table is split in two parts, part A contains all cases where no metabolites are annotated within enzymes.

**A. No metabolites annotated within enzymes**
PMC3406255: "We also determined that **pyruvate dehydrogenase**, turnover of the TCA cycle, anaplerosis and de novo glutamine and glycine synthesis contributed significantly to the ultimate disposition of glucose carbon."
PMC3406255: "The 4-5 doublet in glutamate and glutamine carbon 4 is derived from [1,2-13C]acetyl-CoA produced from [U-13C]glucose, demonstrating that glucose was metabolized to acetyl-CoA via **pyruvate dehydrogenase** (PDH)."
PMC3406255: "Detection of glycine in the breast metastasis is of particular interest since **phosphoglycerate dehydrogenase**, an enzyme in the serine/glycine biosynthesis pathway, is commonly over-expressed in human breast adenocarcinoma, is amplified at the genomic level in a subset of these tumors, and is essential for tumor growth in a human breast cancer xenograft model [29]."
PMC3406255: "For example, mutations in **isocitrate dehydrogenase** isoforms 1 and 2 are commonly found in low-grade gliomas and influence intermediary metabolism, including some of the pathways analyzed in this work [44–46]."
PMC4525767: "However, another equally plausible hypothesis is that reductions in the activity of **branched chain ketoacid dehydrogenase** (BCKD) and **tyrosine aminotransferase** (TAT) in states of insulin resistance lead to increased tissue and circulating BCAA and AAA concentrations, respectively [19]."
PMC4525767: "Metformin has previously been shown to reduce gluconeogenesis and hepatic glucose production [56] and this effect may be at least partially mediated via reduction in glutamic acid/glutamate concentration, although gluconeogenesis is highly regulated by the rate-limiting enzyme, **phosphoenolpyruvate carboxykinase**."
PMC4525767: "There is also recent evidence demonstrating that metformin suppresses gluconeogenesis by inhibiting **mitochondrial glycerol phosphate dehydrogenase** [57]."
PMC5287439: "N1-methylinosine is found 3' adjacent to the anticodon at position 37 of eukaryotic tRNAs and is formed from inosine by a specific **S-adenosylmethionine-dependent methylase**. 25"
PMC6224486: "Moreover, a tryptophan catabolic enzyme, **indoleamine 2,3-dioxygenase**, has been reported as a central driver of malignant development and progression (39)."
PMC6316856: "Serum glucose, HbA1c, triglycerides, total cholesterol, LDL and HDL-cholesterol, **alanine aminotransferase** (ALT), **aspartate aminotransferase** (AST), **gamma-glutamyl transpeptidase** (γ GT), creatinine, uric acid, vitamin D, folic acid, ferritin, c-reactive protein, thyroxin and thyroid-stimulating hormone were measured in a certified clinical laboratory, using standard protocols."
PMC6875299: "In the glucose-alanine cycle pathway, **glutamate dehydrogenase** in muscle catalyzes the binding of α -ketoglutaric acid to ammonia to form glutamate, followed by glutamate catalyzed by **alanine aminotransferase**; pyruvic acid forms alpha-ketoglutarate and alanine [30]."

Table S2: Example output from metabolite NER[S2] (underlined) and enzyme NER (in bold). All sentences (in addition to the table in the main manuscript) for all 18 articles used for the evaluation (see Materials and Methods). Correct annotations are indicated in green, false positive metabolite annotations within enzymes in red, and false negative and other false positive metabolite annotations in orange. Note: formatting (e.g., superscript) is removed after processing with Auto-CORPus[S3] before NER. Table is split in two parts, part B contains all cases where metabolites are falsely contained within enzymes.

**B. Annotation of metabolite within an enzyme, false positive metabolite annotations can be filtered out**
PMC3406255: "The labeling patterns in glutamate and glutamine were similar at each carbon, demonstrating that glutamate was converted to glutamine by the enzyme **glutamine synthetase** (GS)."
PMC3406255: "**Pyruvate dehydrogenase** (PDH), **citrate synthase** (CS), complete turnover of the TCA cycle, anaplerosis, and synthesis of glutamate and glutamine from glucose were evident in all eleven tumors."
PMC3406255: "Anaplerosis is defined as the anaplerotic flux relative to **citrate synthase** activity."
PMC3406255: "As measured by 13C NMR isotopomer analysis, the anaplerotic flux in every tumor approached or exceeded entry of carbon into the TCA cycle via **citrate synthase**, with a total anaplerotic flux of 1.25 $\pm$ 0.43 (mean and SD) relative to **citrate synthase** for the five GBMs infused at 8g/hr (Table 1), indicating robust anaplerosis in these tumors."
PMC3645730: "In fact, higher levels of **ceramide synthases** and ceramide were detected in breast cancer tissues compared with those in adjacent normal ones [42]."
PMC3645730: "Recently, Nagahashi and colleagues showed the importance of **sphingosine kinase** 1-produced S1P to breast cancer-induced hemangiogenesis and lymphangiogenesis in a mouse model [44]."
PMC5073280: "Undiluted serum sample (25 μL) and 100 μL of 2 mM hydrogen peroxide was added to freshly prepared reaction mixture consisting of 755 μL of 0.1 M sodium phosphate buffer (pH 7.0), 100 μL of 2 mM nicotinamide adenine dinucleotide phosphate (NADPH), 10 μL **glutathione reductase** (2 μL in 500 μL buffer) and 10 μL reduced glutathione (9.22 mg/mL) kept in cuvette of 1 mL capacity."
PMC5073280: "The specific activity of **Glutathione peroxidase** (GPx) was expressed as μmoles of NADPH oxidized/min/mg protein as explained by Mills 1959 16."
PMC5073280: "**Superoxide dismutase** (SOD) activity was determined following standard method 18."
PMC5073280: "Similar trend was also observed in **non-enzymatic glutathione peroxidase** activity (Fig. 2D)."
PMC5073280: "Levels of Protein (A), **Lipid Peroxidase** (B), **Catalase** (C), **Glutathione Peroxidase** (D), **Superoxide Dismutase** (E), and Reactive Oxygen Species (ROS) (F) in study groups are presented in box and whisker plots (**, ***p value $<$ 0.01, and $<$ 0.005, respectively, of Wilcoxon-Mann-Whitney test)."
PMC5073280: "ARDs not only showed the highest levels of oxidative stress in terms of ROS and **lipid peroxidase** activity but also had the lowest proportion of **catalase**, **superoxide dismutase** and **glutathione peroxidase** activity."
PMC5287439: "As a membrane constituent, sphingomyelins are implicated in transmembrane signaling and are generated from phosphatidylcholine and ceramide by **sphingomyelin synthase**, the knockout of which leads to mitochondrial dysfunction and reduced insulin release."
PMC6150202: "For enhancing therapeutic effect, thiopurines are very often used in co-medication with other therapeutics, such as corticosteroids (prednisone, PRE, strong anti-inflammatory agent), nonsteroidal anti-inflammatory drugs (mesalazine, 5-amino-salicylic acid, MSL, potent scavenger of reactive oxygen species), and enzyme inhibitors (allopurinol, ALP, inhibitor of **xanthine oxidase**)."
PMC6224486: "MG00/1846Z,9Z,12Z,15Z/00, another metabolite in the panel, is a monoacylglyceride that can be broken down by **monoacylglycerol lipase**."
PMC6224486: "In the present study, the level of monoacylglyceride was low probably due to the high level of **monoacylglycerol lipase**, which has been shown to promote hepatocellular carcinoma and colorectal cancer (24, 25)."
PMC6708594: "Once inside the cell, fludarabine is sequentially phosphorylated to the monophosphate (F-ara-AMP), diphosphate (F-ara-ADP), and triphosphate (F-ara-ATP) forms by **deoxycytidine kinase** (dCK), **adenylate kinase** (AK), and **nucleoside diphosphate kinase** (NDK), respectively [32]."

References

(S1) Islamaj, R.; Kwon, D.; Kim, S.; Lu, Z. TeamTat: a collaborative text annotation tool. *Nucleic Acids Research* **2020**, *48*, W5–W11.

(S2) Yeung, C. S.; Beck, T.; Posma, J. M. MetaboListem and TABoLiSTM: Two Deep Learning Algorithms for Metabolite Named Entity Recognition. *Metabolites* **2022**, *12*, 276.

(S3) Beck, T.; Shorter, T.; Hu, Y.; Li, Z.; Sun, S.; Popovici, C. M.; McQuibban, N. A. R.; Makraduli, F.; Yeung, C. S.; Rowlands, T.; Posma, J. M. Auto-CORPus: A Natural Language Processing Tool for Standardising and Reusing Biomedical Literature. *Frontiers in Digital Health* **2022**, *4*, 788124.
